# Whole genome sequencing and molecular detection of potato virus X in Bangladesh

**DOI:** 10.1371/journal.pone.0322935

**Published:** 2025-05-08

**Authors:** Md. Shafiqul Islam, Md. Akash Ahammed, Fahmida Akhter, Mosaddiqur Rahman, Md. Mosharraf Hossain Molla

**Affiliations:** 1 Tuber Crops Biotechnology and Seed Production Section, Tuber Crops Research Centre, Bangladesh Agricultural Research Institute, Gazipur, Bangladesh; 2 Breeder Seed Production Centre, Bangladesh Agricultural Research Institute, Debiganj, Panchagarh, Bangladesh; ICAR - Indian Agricultural Research Institute, INDIA

## Abstract

Potato Virus X (PVX) is a significant viral pathogen affecting potato (*Solanum tuberosum*) crops globally, yet its molecular characterization in Bangladesh remains limited. This study presents the first whole genome sequence (WGS) and molecular analysis of PVX isolated from potato plants in Gazipur, Bangladesh. Initial virus detection was performed using DAS-ELISA on symptomatic potato leaves, followed by RT-PCR targeting the coat protein (CP) gene, which confirmed PVX presence in ‘Patnai’ and ‘Challisha’ potato varieties through a 562 bp amplicon. The WGS of the Patnai-PVX isolate was determined to be 6,435 nucleotides long and deposited in GenBank (accession: PQ527059). Genome analysis identified five major open reading frames encoding the RNA-dependent RNA polymerase (RdRp), triple gene block proteins (TGBp1, TGBp2, TGBp3), and CP. Basic Local Alignment Search Tool X (BLASTX) analysis revealed high sequence similarity with PVX isolates from neighboring regions, suggesting evolutionary conservation. Mutation analysis identified 265 SNPs, predominantly synonymous mutations, indicating maintained protein-coding integrity despite genetic variation. Fewer non-synonymous mutations were detected, potentially affecting viral protein functions and pathogenicity. Phylogenetic analysis based on the entire genome sequence placed the Bangladeshi isolate (PQ527059.1) in a well-supported clade (bootstrap value 99%) with isolates from Peru (MT752634.1, MT752612.1, MT752621.1), highlighting potential international transmission routes while also exhibiting unique genetic markers indicative of regional specificity. This comprehensive molecular characterization enhances understanding of PVX genetic diversity and evolution in Bangladesh, providing valuable insights for developing effective virus management strategies in potato cultivation.

## Introduction

Whole-genome sequencing (WGS) has revolutionized virology by offering an in-depth perspective of viral genomes and their evolutionary trajectories. In the case of PVX, a widespread and economically significant plant virus, this technology provides critical insights into its genetic structure and transmission dynamics [1].

PVX is a member of the *Potexvirus* genus in the family Alphaflexiviridae [2,3] comprising a positive-sense, single-stranded RNA genome of approximately 6.4 kb. The genome encodes five open reading frames (ORFs), including the *CP* gene, which plays a pivotal role in viral assembly and host interaction [4–7]. PVX was first identified in the United Kingdom by Smith 1931 [8], has since become one of the most globally prevalent viruses affecting potato crops, causing significant economic losses due to reduced tuber yield and compromised plant health [9]. Transmission of PVX primarily occurs through mechanical means, including contact between infected and healthy plants, farming tools, and contaminated surfaces [10–12]. Additionally, infected potato tubers propagate the virus, enabling rapid spread across fields[2]. PVX infections can reduce potato yields by up to 10–40% [13,14] with greater losses observed when co-infected with other viruses such as Potato Virus Y (PVY), which triggers synergistic effects that can devastate crops by up to 80% [14].

Potatoes are a critical staple crop worldwide, with global production reaching approximately 375 million tonnes in 2022, with China and India leading production. Within Asia, Bangladesh ranks as the fourth-largest potato producer and is among the top 15 globally [15]. However, PVX poses a significant threat to Bangladesh’s potato sector, which is vital for both food security and economic stability. Despite its importance, molecular characterization of PVX isolates from Bangladesh has been limited, particularly in understanding the genetic diversity within local strains. Effective detection of PVX is essential for managing its spread and mitigating its impact. Traditional methods like Enzyme-Linked Immunosorbent Assay (ELISA) are commonly used for initial screening due to their sensitivity. However, molecular techniques such as Polymerase Chain Reaction (PCR) and sequencing are indispensable for confirming the virus’s presence and revealing genetic diversity [16].

WGS and *CP* gene sequences of PVX isolates worldwide have identified significant genetic variation [17], playing a crucial role in viral adaptation, host range, and virulence [18–20]. This genetic variation arises from high mutation rates, recombination events, and selection pressures [18]. These factors enable PVX to adapt to different environmental conditions and host plants, enhancing its ability to infect a wide range of hosts and increasing its virulence. The accumulation of genetic changes allows PVX to evolve rapidly, overcoming host defenses and establishing successful infections. For example, substitution mutations in the *CP* gene have been associated with overcoming resistance genes like *Rx* [21].

Phylogenetic analyses of global PVX isolates have revealed distinct clades, often linked to geographical origins, suggesting localized evolutionary trends and potential common ancestry. In Bangladesh, seven potato viruses have been reported, including Potato Leafroll Virus (PLRV), Potato Virus Y (PVY), Potato Virus X (PVX), and others. PVX, along with PLRV and PVY, are the most prevalent, contributing to yield losses [16,22,23]. However, there remains limited molecular data on PVX isolates from Bangladesh.

This study aims to fill this gap by detecting and characterizing PVX from potato plants in Gazipur, Bangladesh, using ELISA, PCR, and WGS. Furthermore, the study provides a phylogenetic comparison of the Bangladeshi isolate with global PVX strains, shedding light on its evolutionary relationships. In addition to sequencing the whole genome, the study identified key open reading frames (ORFs) coding for viral proteins, consistent with those reported in previous studies. Mutation analysis revealed several single nucleotide polymorphisms (SNPs) in comparison to reference PVX genomes, which could influence the virus’s pathogenicity or adaptation. Using BLAST analysis, the sequences were aligned with global PVX isolates, confirming high sequence similarity with isolates from neighboring regions and uncovering unique genetic variations in the Bangladeshi PVX strain. Understanding the genetic diversity, biology, and mutation profile of PVX is crucial for developing effective management strategies. By investigating the whole genome of PVX from Gazipur, this study offers valuable insights into the virus’s evolutionary history, genetic makeup, and potential targets for control measures, contributing to the ongoing efforts to safeguard potato production from this persistent threat.

## Materials and methods

### Sample collection

Potato leaf samples were collected from 14 symptomatic potato plants from the research field of the Tuber Crops Biotechnology and Seed Production Section, Tuber Crops Research Centre (TCRC), Bangladesh Agricultural Research Institute (BARI), Gazipur-1701, Bangladesh, during the growing season. No additional permits were required as the research was conducted within the institute’s jurisdiction. The samples, including varieties such as Sadaguti, Borjam Alu, Ausha, Indurkani, Lalpakri, Asterix, BARI Alu-7, Ausha (2), BARI Alu-25, BARI Alu-36, BARI Alu-53, Dohazari, Patnai, and Challisha were carefully transported to the laboratory in sterile, zip-lock plastic bags placed in an ice box to maintain a low temperature (S1 Table). Upon arrival, they were stored at -20 °C until further processing.

### DAS-ELISA assay for initial screening

To detect the presence of PVX, a Double Antibody Sandwich Enzyme-Linked Immunosorbent Assay (DAS-ELISA) was performed [24] using a commercial PVX-specific ELISA kit (PVX Complete kit 96, Bioreba, Switzerland). Briefly, wells were coated with specific IgG antibodies diluted in coating buffer, incubated at 30 °C for 4 h, and then washed to remove unbound antibodies. Plant extracts prepared in extraction buffer were added to the wells, incubated overnight at 4 °C, and washed again. An enzyme-labeled antibody conjugate was then applied and incubated at 30 °C for 5 h, followed by further washing. A para-nitrophenylphosphate substrate solution was added to initiate a color reaction, with color development monitored at 405 nm after 60 min. Positive and negative controls, provided with the PVX Complete kit 96, were included in each assay to ensure reliability and accuracy. Positive samples were identified based on the intensity of color change, indicating the presence of PVX.

### RNA isolation

Total RNA was extracted from plant tissue samples using a protocol based on the SV Total RNA Isolation System (Promega, USA). Plant material was initially pulverized in liquid nitrogen using a sterile mortar and pestle. The homogenized tissue (30 mg) was immediately transferred to RNA Lysis Buffer (175 µ L) and diluted with 350 µ L of dilution buffer. Following inversion mixing, the lysate was centrifuged (12,000 × g, 10 min, room temperature) to remove cellular debris. The clarified supernatant was transferred to a fresh microcentrifuge tube and combined with 95% ethanol (200 µ L) to optimize binding conditions. The resulting mixture was applied to a spin column and centrifuged (12,000 × g, 1 min). After discarding the flow-through, the membrane-bound RNA was subjected to a wash step using ethanol-supplemented wash solution. Genomic DNA contamination was eliminated through on-column DNase digestion. The DNase reaction mixture, comprising DNase I enzyme, Yellow Core Buffer, and MnCl₂, was applied directly to the membrane and incubated at ambient temperature for 15 min. Following DNase inactivation with 200 µ L stop solution and subsequent membrane washing steps, purified RNA was eluted in 100 µ L nuclease-free water. RNA samples were stored at –20 °C until further analysis.

### First-strand cDNA synthesis

Reverse transcription was performed using the GoScript™ Reverse Transcription System (Promega, USA). The reaction was initiated by combining total RNA (4 µ L) with oligo(dT) primers in nuclease-free water to a final volume of 5 µ L. This mixture underwent denaturation at 70 °C for 5 min, followed by immediate ice-cooling. The reverse transcription master mix was formulated to contain 4.0 µ L of 5X reaction buffer, 4.0 µ L optimized MgCl₂ (5.0mM), PCR nucleotide mix (1.0 µ L), recombinant RNase inhibitor (20 units), and reverse transcriptase (1.0 µ L), with nuclease-free water added to achieve a final volume of 15 µ L. The complete reaction mixture was subjected to a sequential thermal program consisting of primer annealing (25 °C, 5 min), cDNA synthesis (42 °C, 60 min), and enzyme inactivation (70 °C, 15 min). The resulting cDNA was stored at –20 °C for subsequent applications.

### PCR amplification

PCR was performed on all 14 samples, regardless of their ELISA results, to check for the presence of PVX using specific primers (Table 1) targeting the *CP* gene. PCR reactions were carried out in a 20 µ L volume containing 10 µ L of 2X PCR master mix (EmeraldAmp GT PCR Master Mix, Takara Bio, Japan), 1 µ L of each primer (forward and reverse, at 10 µ M), 2 µ L of cDNA, and nuclease-free water. The thermocycler conditions were as follows: initial denaturation at 95 °C for 5 min, followed by 35 cycles of denaturation at 95 °C for 30 seconds, annealing at 55 °C for 30 seconds, extension at 72 °C for 30 seconds, and a final extension at 72 °C for 5 min. The reaction is then held at 4 °C to prevent degradation of the PCR products, which can be analyzed using gel electrophoresis or other methods to confirm successful amplification of the target sequence. PCR products were analyzed by gel electrophoresis on a 1% agarose gel stained with ethidium bromide. DNA bands were visualized under UV light and photographed.

**Table 1:** PVX detection primer.

Primer	Primer sequence (5′-3′)	Product size (bp)	Reference
**PVX-F**	TAGCACAACACAGGCCACAG	562	[25]
**PVX-R**	GGCAGCATTCATTTCAGCTTC		

### Whole genome sequencing

One PVX-positive sample (Patnai-PVX), identified through PCR, was selected for WGS because it was obtained from one of the widely grown and economically important potato varieties in Bangladesh affected by PVX. Additionally, it exhibited clear PVX-associated symptoms, making it suitable for sequencing, and had a high viral load, as indicated by strong PCR amplification, ensuring reliable genome assembly. Future studies may explore additional samples to assess genetic variability. Available WGS of PVX from the NCBI GenBank database were used to design six sets of overlapping primers (Table 2) to amplify the entire viral genome (S2 Table). These primers were designed to provide adequate coverage and overlap across genomic regions, ensuring accuracy in sequencing and assembly. PCR amplification was performed as described above, and PCR products were purified using the QIAquick PCR Purification Kit (QIAGEN, Germany) before being sent for Sanger sequencing. Each region of the PVX genome was amplified with six sets of overlapping primers, and Sanger sequencing was conducted in both forward and reverse directions for each amplicon. Bidirectional sequencing ensured high-quality sequence data and accuracy in genome assembly. Sequencing reads from both directions were aligned and assembled to produce the WGS. Sequence data were assembled using MEGA 11[26] software for alignment and analysis, allowing for precise assembly of the WGS. BioEdit [27] also inspected and refined nucleotide sequences for consistency. Final adjustments and minor corrections were made manually in Microsoft Word 19 (https://www.microsoft.com) to ensure accuracy. The assembled sequence was subsequently submitted to GenBank with the accession number PQ527059.

**Table 2:** PVX WGS primers.

Primer	Primer sequence (5′-3′)	Product size (bp)	Range	Overlapping Sequence (bp)
**PVX1-F**	GAAAACTAAACCATACACCAC	1034	1-1034	–
**PVX1-R**	AAGAAGAGCTGCATCATAGT			
**PVX2-F**	AAGAAAACTATGATGCAGCTCT	944	1009-1952	25
**PVX2-R**	ATCCAGTGTTTCCAAGGGA			
**PVX3-F**	AGGTGGGAAGATGCTTCATT	1196	1828-3023	124
**PVX3-R**	GGGGTGCTGTCTAACTTTTC			
**PVX4-F**	GACCACAACACCCAAGTGTG	1115	2896-4010	127
**PVX4-R**	ATGTTGCACTCAGTGTTTGCA			
**PVX5-F**	CACCACTGCATACCAGAGGAAAT	1365	3874-5238	136
**PVX5-R**	GTAATTGAAACTAAAGCAAATGA			
**PVX6-F**	AATTCTGAAAAAGTGTACATAGT	1253	5183-6435	55
**PVX6-R**	ATTTATATTATTCATACAATCAAACC			

### Identification of ORFs

The open reading frames (ORFs) in the WGS of Patnai-PVX were identified using the NCBI ORF Finder tool [28]. This tool was employed to predict potential protein-coding regions within the nucleotide sequence. The ORF Finder scanned the genome for start and stop codons, providing a list of potential ORFs based on the genetic code. Once the ORFs were identified, they were further validated by performing BLASTX [29] searches against the NCBI non-redundant protein database (nr). BLASTX allowed for the comparison of the identified ORFs to known protein sequences, ensuring the accurate annotation of viral proteins. Only ORFs with significant similarity to known viral proteins (E-value < 1e-5) and high sequence identity were retained. The ORF corresponding to the *CP* gene was identified from the WGS of PVX. The *CP* gene sequence was extracted and compared with the *CP* gene sequences of three PVX isolates from Bangladesh (GenBank accession numbers: MK587459.1, MF589763.1, JX273242.1). The comparison was performed using the “Align two or more sequences” option of NCBI BLASTN [30]. This tool enabled a direct sequence-to-sequence alignment, calculating percentage identity, query coverage, and E-value to assess the similarity between the query and the subject sequences.

### Mutation analysis and functional annotation

Variant calling was performed using bcftools [31]to identify SNPs by comparing our newly sequenced Patnai-PVX isolate (GenBank accession number: PQ527059.1) against the PVX reference genome (GenBank accession number: NC_011620.1). The identified variants were compiled in a variant call format (VCF) file (S1 File). These variants were annotated using SnpEff [32] with a custom genome database created using the PVX reference genome (GenBank accession number: NC_011620.1) and corresponding gene annotations in GFF format (S2 File). SnpEff classified the variants by functional impact (modifier, low, moderate, high) and mutation type (synonymous, missense, nonsense). The transition to transversion ratio (Ts/Tv) was calculated to determine the prevalence of transition versus transversion mutations. Additionally, the variants were mapped to different genomic regions, including upstream, exonic, and downstream areas, to evaluate their distribution and potential effects on gene regulation and protein-coding sequences.

### Phylogenetic analysis

For phylogenetic analysis, a total of 27 sequences were retrieved from the NCBI GenBank database, representing isolates from diverse geographical regions including Europe, Asia, North America, South America, Africa, and Oceania (S3 Table). The evolutionary history was inferred using the Maximum Likelihood (ML) method [33] implemented in MEGA11 software. The Tamura-Nei substitution model [34] was selected for the analysis. Initial trees for the heuristic search were obtained through Neighbor-Joining [35] and BioNJ algorithms [36], using a matrix of pairwise distances estimated with the Tamura-Nei model. The topology with the superior log likelihood value was selected. Branch support was assessed using 1000 bootstrap replicates, and values are shown as percentages at the nodes. The final dataset contained 6,478 positions.

## Results

**Fig 1:**
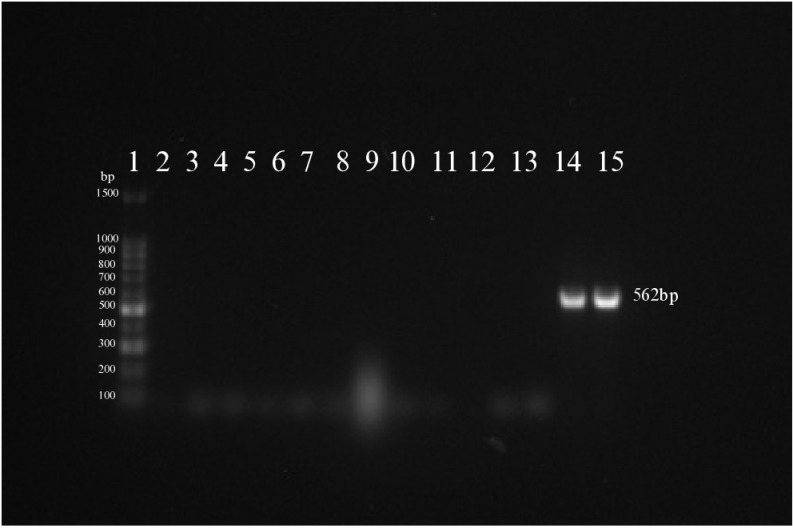
Gel electrophoresis analysis of PCR products for PVX detection in potato samples. PCR results using a 100 bp DNA ladder as a size marker (Lane 1). Lanes 2–15 represent potato samples: 2 - Sadaguti; 3 - Borjam Alu; 4 - Ausha; 5 - Indurkani; 6 - Lalpakri; 7 - Asterix; 8 - BARI Alu-7; 9 - Ausha (2); 10 - BARI Alu-25; 11 - BARI Alu-36; 12 - BARI Alu-53; 13 - Dohazari; 14 - Patnai; 15 - Challisha. Samples Patnai and Challisha (Lanes 14 and 15) show a distinct band at 562 bp, indicating the presence of PVX, while no bands are observed in the other lanes.

In this study, out of the 14 potato leaf samples tested, 2 (Patnai and Challisha) were confirmed positive for PVX using the ELISA, demonstrating the initial detection of the virus. The ELISA-positive and negative samples were further validated using PCR, specifically targeting the *CP* gene of PVX. PCR amplification produced a 562 bp fragment in 2 samples, Patnai and Challisha, confirming the presence of PVX in these samples (Fig 1). This combination of serological and molecular techniques underscored the reliability of virus identification, enhancing confidence in the diagnostic process. Following confirmation, the sample (Patnai-PVX) from Patnai variety was selected for entire genome sequencing. Patnai is a regional variety highly valued in local agriculture and popular among farmers. Efforts will be undertaken to make this variety virus-free using advanced technologies such as meristem culture, thermotherapy, and CRISPR-based gene editing [37–39].

**Fig 2:**
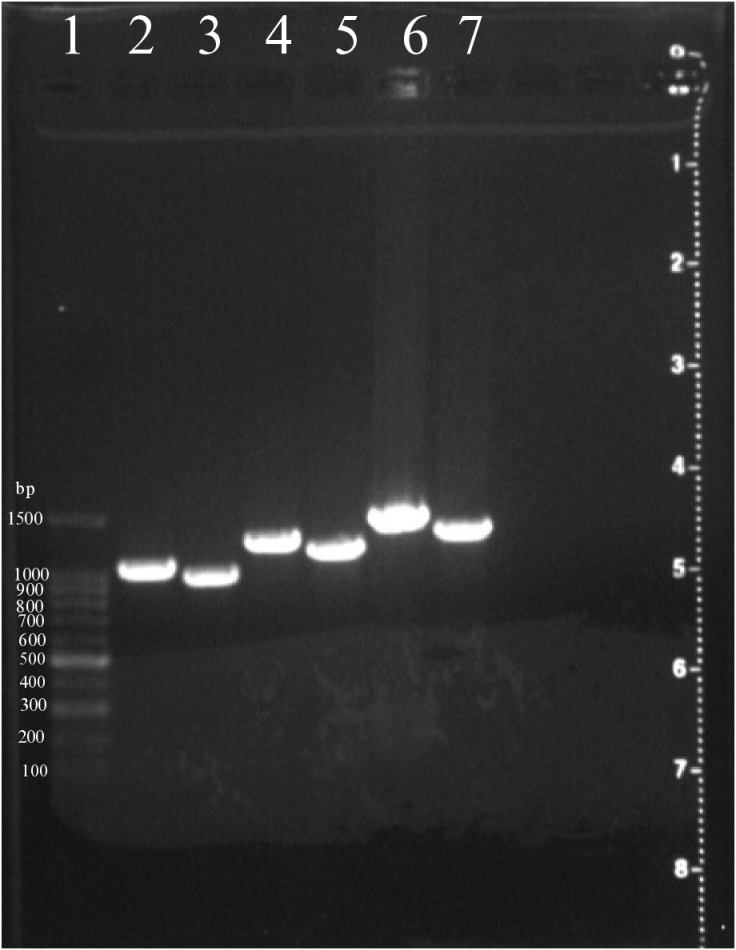
Gel electrophoresis analysis of PCR-amplified fragments for the whole genome of PVX using six primer sets. Image showing PCR amplification of various regions of the PVX genome. Lane 1 contains a 100 bp DNA ladder, used as a size reference. Lanes 2 through 7 contain PCR products from different PVX-specific primer sets, generating distinct bands corresponding to the expected amplicon sizes: PVX1 (1034 bp), PVX2 (944 bp), PVX3 (1196 bp), PVX4 (1115 bp), PVX5 (1365 bp), and PVX6 (1253 bp).

The PCR products from six sets of primers were visualized using gel electrophoresis to confirm the presence and expected size of the amplified PVX genome fragments (Fig 2). The clear, specific bands with expected sizes confirm the successful amplification of each genome fragment, suitable for subsequent sequencing steps. No nonspecific bands or primer dimers were observed, indicating high specificity of the primer pairs for the PVX genome.

The sequencing process yielded the whole PVX genome, consisting of 6,435 nucleotides, and the resulting sequence was deposited in the GenBank database with the accession number PQ527059. The WGS of the Patnai-PVX isolate was analyzed for the presence of open reading frames (ORFs) using the NCBI ORF Finder, a standard bioinformatics tool. BLASTX analysis confirmed the presence of five major ORFs encoding the RdRp, three triple gene block proteins (TGBp1, TGBp2, TGBp3), and the CP (S3–S7 Files). These ORFs correspond to the typical genomic structure of Potato Virus X (PVX), consistent with previously published PVX isolates [17,40,41].

BLAST analysis of the PVX *CP* gene sequence was performed against three *CP* gene sequences from Bangladesh. The query sequence showed 98.74% identity with the PVX strain Bogra (MK587459.1), 99.28% identity with isolate PVX-Munshiganj (MF589763.1), and 98.32% identity with isolate PVX-BD-B1 (JX273242.1). Query coverage ranged from 97% to 100%, with all E-values being 0.0, indicating highly significant matches (S8 File).

The mutation analysis of the PVX genome identified 265 single nucleotide polymorphisms (SNPs), with no insertions, deletions, or multi-allelic variants detected. Most variants (79.9%) were classified as having a modifier impact, meaning they are unlikely to affect gene function significantly. low impact variants accounted for 17.27%, while moderate impact variants were 2.75%. Only one high impact variant, a nonsense mutation (T → A) was identified at position 1988 in the *RdRp* gene of PVX, resulting in a premature stop codon at position 635 (p.Leu635*) (S9 File). This mutation is likely to disrupt the production of a functional RdRp enzyme, which is essential for viral RNA replication. As a result, the mutation could significantly impair the virus’s replication capability and infectivity. Further studies are needed to confirm the biological impact of this mutation on PVX functionality. Most mutations (85.9%) were synonymous, meaning they did not change the protein sequence, while 13.7% were missense, causing amino acid changes. The transition/transversion ratio was 6.16, showing a predominance of transitions. Regionally, 66.39% of the variants were upstream of coding regions, 20.09% were in exons, and 13.29% were downstream (S10 File). This high proportion of synonymous mutations suggests the virus maintains protein function despite a high mutation rate.

**Fig 3:**
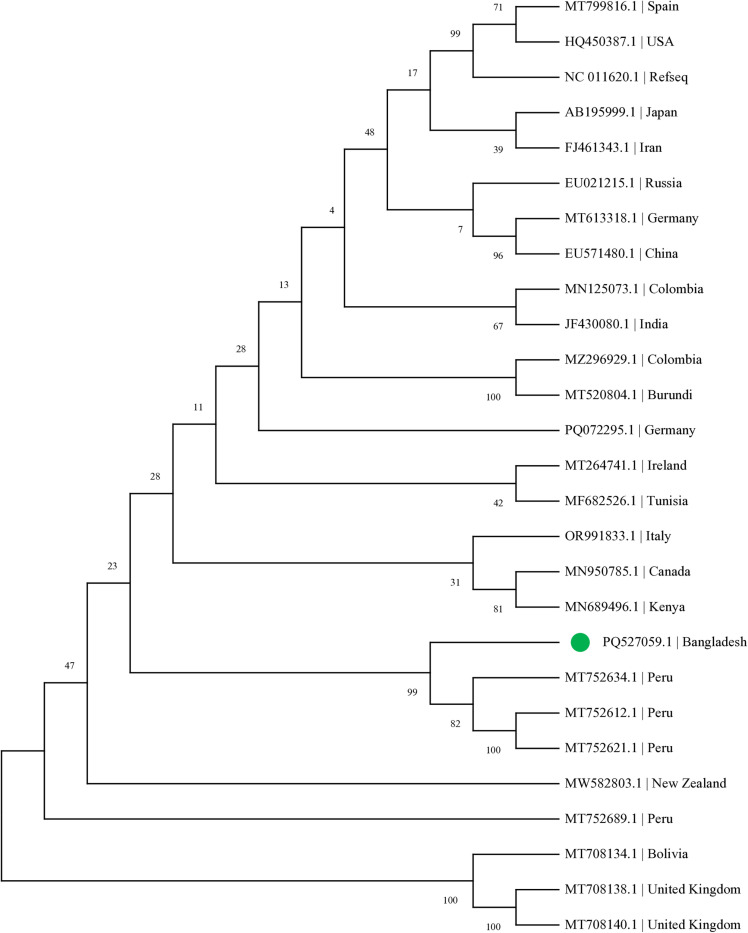
Maximum Likelihood phylogenetic tree of viral isolates from different geographical regions. The Bangladesh isolate (PQ527059.1) clustered with Peru isolates with 99% bootstrap support. Other notable clusters include the United Kingdom-Bolivia clade (100% bootstrap support) and the Spain-USA clade (99% bootstrap support). The tree is drawn to scale, with branch lengths measured in the number of substitutions per site. All positions containing gaps and missing data were eliminated from the analysis. The reference sequence (NC_011620.1) is included. Country of origin for each sequence is indicated after the accession number. The analysis was conducted using MEGA11 software, and the tree with the highest log likelihood (-33321.23) is shown. Scale bar represents the number of nucleotide substitutions per site.

The Maximum Likelihood phylogenetic analysis produced a tree (Fig 3) with a log likelihood value of -33321.23. The Bangladesh isolate (PQ527059.1) formed a distinct clade with three Peru isolates (MT752634.1, MT752612.1, MT752621.1), supported by a high bootstrap value of 99%. This cluster was separate from the Asian isolates, including those from Japan (AB195999.1), Iran (FJ461343.1), China (EU571480.1), and India (JF430080.1). Several other well-supported clusters were observed, including a group of UK and Bolivian isolates (bootstrap value 100%), and a cluster containing isolates from Canada (MN950785.1) and Kenya (MN689496.1) with 81% bootstrap support. The European isolates, including those from Spain (MT799816.1) and USA (HQ450387.1), formed a separate cluster with 99% bootstrap support.

## Discussion

This study presents the first comprehensive molecular characterization and WGS of PVX from Bangladesh, providing crucial insights into the genetic composition and diversity of PVX in the region. The successful detection of PVX using both serological (DAS-ELISA) and molecular (RT-PCR) methods demonstrates the effectiveness of combining multiple diagnostic approaches, a strategy supported by previous studies for reliable virus detection [42,43].

The WGS of Patnai-PVX (6,435 nucleotides) aligns with the typical genome size reported for PVX isolates worldwide, falling within the expected range of 6.4–6.5 kb [2,17]. The genomic organization, comprising five major ORFs encoding the RdRp, TGBp1, TGBp2, TGBp3, and CP, corresponds to the canonical structure of potexviruses [4,5,44]. This conservation of genomic architecture suggests maintaining essential viral functions despite geographic isolation. The mutation analysis revealed interesting patterns in the genetic variation of the Bangladeshi PVX isolate. The identification of 265 SNPs, predominantly synonymous mutations (85.9%), indicates strong purifying selection pressure maintaining functional protein sequences [40,45,46]. The high transition/transversion ratio (6.16) observed in our study is characteristic of RNA virus evolution and suggests a bias toward transitions, possibly due to the biochemical nature of RNA mutations [47,48]. The high mutation rate observed in PVX, as in other RNA viruses, is attributed to the absence of proofreading activity in RNA-dependent RNA polymerases (RdRp) [49,50]. This contributes to genetic variability, allowing PVX to adapt to diverse hosts and environmental conditions, while presenting challenges for disease management and resistance breeding programs. For instance, the mutation rate of Tobacco mosaic virus (TMV), another plant RNA virus, has been estimated at 0.10–0.13 mutations per genome, based on the detection of mutants lethal for cell-to-cell movement [51,52]. Similar plant viruses such as Potato Virus Y (PVY) and Potato Leafroll Virus (PLRV) exhibit high mutation rates [53,54]. Such mutation rates underscore the evolutionary potential of RNA viruses and the challenges they pose for effective control strategies.

Phylogenetic analysis revealed interesting patterns of evolutionary relationships among the viral isolates from different geographical regions. The Bangladeshi isolate (PQ527059.1) formed a well-supported clade (bootstrap value 99%) with isolates from Peru (MT752634.1, MT752612.1, MT752621.1), suggesting potential international transmission routes. This clustering pattern, where geographically distant isolates share close genetic relationships, indicates that viral dispersal may be influenced by factors beyond geographical proximity, such as international trade networks or agricultural exchange practices [55]. The tree topology showed several distinct clusters with varying levels of geographic correlation. For instance, the Peru isolates formed a monophyletic group with strong bootstrap support (82–100%), indicating local evolution and adaptation. Similarly, the United Kingdom isolates (MT708138.1, MT708140.1) clustered together with a Bolivian isolate (MT708134.1) with maximum bootstrap support (100%), suggesting a common evolutionary origin despite their geographical separation.

The presence of moderate-impact variants (2.75%) and one high-impact variant in the genome warrants attention, as these could potentially influence viral fitness or host adaptation. Previous studies have shown that even single amino acid changes in viral proteins can affect pathogenicity and host range [56–59]. The concentration of variants in upstream regions (66.39%) suggests possible regulatory adaptations, which could influence viral gene expression patterns.

The detection of PVX in the widely grown potato varieties ‘Patnai’ and ‘Challisha’ has significant implications for potato cultivation in Bangladesh. Although most plants displayed viral symptoms, they tested negative for PVX because our testing was specifically targeted to PVX, and the symptoms observed could have been caused by other viruses not included in this test panel. This highlights the potential for multiple viral infections contributing to the symptoms in these plants, suggesting the need for broader testing and improved virus management strategies in potato cultivation. These varieties ‘Patnai’ and ‘Challisha’ are important for local agriculture, and their susceptibility to PVX infection highlights the need for enhanced disease management strategies. Similar concerns have been raised in other potato-growing regions where PVX infection can reduce yields by 10–40% [60]. From a methodological perspective, our combined approach using DAS-ELISA and RT-PCR for initial detection, followed by WGS, provides a robust framework for virus surveillance. Several researchers have recommended this multi-tiered diagnostic approach for comprehensive plant virus detection and characterization [24]. The high sequence similarity with other Bangladeshi isolates (PVX-Munshiganj, PVX-BD-B1, and Bogra) suggests limited genetic diversity within the country’s PVX population. This could indicate either recent introduction events or strong selection pressures maintaining certain genetic variants.

The WGS and genetic characterization of local PVX strains can improve diagnostic tools, making virus detection more specific and accurate [61–64]. Advanced technologies, such as CRISPR-Cas systems can leverage the WGS findings from this study for precise detection and targeted virus suppression [65–69].Insights into the genetic diversity of PVX may also support breeding programs aimed at developing virus-resistant potato varieties. Additionally, identifying conserved genomic regions could lead to broad-spectrum resistance strategies. The WGS facilitates the classification of pathotypes based on their ability to overcome potato resistance genes. This information is crucial for developing resistance management strategies and monitoring the potential spread of high-risk isolates, such as Rx-breaking strains, which could have significant agricultural impacts if introduced to new regions [70]. Understanding the geographic distribution of PVX strains allows for tailored management strategies. By identifying which strains are prevalent in specific areas, agricultural authorities can implement targeted control measures. For instance, regions with high incidences of virulent strains may require stricter quarantine protocols for seed potato imports to prevent further spread [71].

Future research should investigate the biological significance of non-synonymous mutations to determine their impact on viral fitness and pathogenicity. Additionally, studies should explore host-pathogen interactions in Bangladeshi PVX strains to identify potential resistance mechanisms. Long-term monitoring of viral evolution will be crucial for detecting emerging variants and understanding their epidemiological implications. Furthermore, assessing the influence of agricultural practices on PVX transmission and genetic diversity can help develop targeted disease management strategies.

## Conclusion

This study presents the first WGS and molecular characterization of Potato Virus X from Bangladesh (Patnai-PVX), comprising 6,435 nucleotides with five major ORFs. The analysis revealed 265 SNPs, with predominantly synonymous mutations, indicating strong conservation of functional viral proteins. High sequence similarity (98–99%) of the *CP* gene with regional isolates indicates potential local evolution patterns of PVX in Bangladesh. The successful implementation of combined serological and molecular detection methods, coupled with WGS, establishes a robust framework for virus surveillance. These findings will contribute significantly to understanding PVX diversity by identifying conserved genomic regions essential for developing broad-spectrum resistance strategies and enhancing diagnostic tools. Insights from the genetic diversity of PVX can also inform resistance breeding programs aimed at developing virus-resistant potato varieties. Additionally, WGS data allow for the classification of pathotypes, aiding in monitoring and managing the potential spread of high-risk isolates. Together, these efforts lay a foundation for targeted disease management strategies to improve potato cultivation in Bangladesh and neighboring regions.

## Supporting information

S1 Table Sample details, including potato varieties. (XLSX)

S2 Table Information on whole genome sequences used for primer design. (XLSX)

S3 Table Information on sequences used for phylogenetic analysis. (XLSX)

S1 File VCF file containing identified genomic variants. (VCF)

S2 File Gene annotation file in GFF format. (GFF)

S3 File BLASTX results for identified ORF1. (TXT)

S4 File BLASTX results for identified ORF2. (TXT)

S5 File BLASTX results for identified ORF3. (TXT)

S6 File BLASTX results for identified ORF4. (TXT)

S7 File BLASTX results for identified ORF5. (TXT)

S8 File CSV file summarizing BLASTX search results. (CSV)

S9 File Annotated variant file with identified high-impact mutations. (VCF)

S10 File SNP annotation summary generated by SnpEff. (HTML)
